# Community-based primary health care for older adults: a qualitative study of the perceptions of clients, caregivers and health care providers

**DOI:** 10.1186/s12877-015-0052-x

**Published:** 2015-04-30

**Authors:** Claire Lafortune, Kelsey Huson, Selena Santi, Paul Stolee

**Affiliations:** School of Public Health and Health Systems, University of Waterloo, 200 University Avenue West, Waterloo, ON N2L 3G1 Canada; Institutional Research, University of Waterloo, 200 University Avenue West, Waterloo, ON N2L 3G1 Canada

**Keywords:** Primary care, Community-based care, Seniors, Health care providers, Chronic illness, Caregivers

## Abstract

**Background:**

Older persons are often poorly served by existing models of community-based primary health care (CBPHC). We sought input from clients, informal caregivers, and health care providers on recommendations for system improvements.

**Methods:**

Focus group interviews were held with clients, informal caregivers, and health care providers in mid-sized urban and rural communities in Ontario. Data were analyzed using a combination of directed and emergent coding. Results were shared with participants during a series of feedback sessions.

**Results:**

An extensive list of barriers, facilitators, and recommended health system improvements was generated. Barriers included poor system integration and limited access to services. Identified facilitators were person and family-focused care, self-management resources, and successful collaborative practice. Recommended system improvements included expanding and integrating care teams, supports for system navigation, and development of standardized information systems and care pathways.

**Conclusions:**

Older adults still experience frustrating obstacles when trying to access CBPHC. Identified barriers and facilitators of improved system integration aligned well with current literature and Wagner’s Chronic Care Model. Additional work is needed to implement the recommended improvements and to discern their impact on patient and system outcomes.

**Electronic supplementary material:**

The online version of this article (doi:10.1186/s12877-015-0052-x) contains supplementary material, which is available to authorized users.

## Background

The growing number of individuals aged sixty-five years and older has led to increased recognition of the need for health care reform [1-3]. As older adults often experience multiple, complex chronic illnesses and functional disabilities, the use of a variety of health services and care providers is required. This makes it difficult to coordinate and integrate care [4-6], resulting in more negative health outcomes, greater use of emergency and acute services, and overall higher health care costs [2,7-11]. Although older adults are the highest users of the health care system [12], their complex care requirements are not well served by existing models of care [1-4,13].

Community-based primary health care (CBPHC) is intended to provide first-contact health services to ensure continuity of care, ease of movement across the system, and improved system integration [3]. However, Canada’s health care system is characterized by fragmentation of services [4,14], with deficits in transitional care and coordination [15-20], and limited efforts to engage or support patients to manage their conditions [21]. Fragmentation of the health care system is particularly challenging for older adults [14].

To inform efforts to improve CBPHC for the older population, and to guide related research, we sought to understand the experiences of CBPHC users, family caregivers, and health care providers from various care settings. This study was performed to answer the questions ‘“What are the barriers and facilitators that older adults encounter when trying to access community-based primary care?’” and ‘“What system improvements would older adults recommend to overcome these barriers and strengthen these facililtators?’”.

## Methods

We undertook a qualitative consultation process involving focus group interviews (and one individual interview) with older CBPHC clients, informal caregivers, and primary health care providers.

### Sampling

A stratified purposive sampling strategy [22] was used. Focus group participants were recruited through phone calls to relevant provider agencies, e-mails sent by key agency contacts, and information posters displayed in local libraries, senior and community centres, and retirement homes. Interested participants were asked to contact the research group to review the eligibility criteria and to facilitate scheduling for the focus group sessions. Criteria for client participation included those over the age of 65 who had received one or more forms of CBPHC, defined as the ‘“broad range of primary prevention (including public health) and primary care services within the community, including health promotion and disease prevention; the diagnosis, treatment, and management of chronic and episodic illness; rehabilitation support; and end-of-life care’” [23], in the previous month, who were English speaking, and who had not been diagnosed with any form of dementia or cognitive impairment. Informal caregivers were recruited if they were English speaking, and had experience caring for family members over the age of 65 who had received one or more forms of CBPHC in the previous month. Inclusion criteria for health care providers (including frontline and administrative personnel) were that they could speak English and that they had provided primary health care services to clients over the age of 65 years. Health care providers could reflect both on their professional roles and on their personal experiences as informal caregivers.

### Data collection

A total of seven focus group interviews, four with health care providers and three with clients and informal caregivers, and one individual informal caregiver interview were held. Focus groups were used in order to obtain rich data on multiple participant views regarding the same topic. There was one individual interview performed because the informal caregiver was interested in participating but was unable to attend the scheduled focus group time. In total, 28 clients and informal caregivers and 20 health care providers participated in the study (see Table 1 for participant characteristics). Interviewed providers included community case managers, occupational therapists, physicians, a registered dietician, a health promoter, a physiotherapist, a social worker, a nurse practitioner, a chiropodist, a hospice coordinator, and a registered practical nurse. Interviews were conducted in urban and rural communities in Southwestern Ontario and lasted between one and two hours. All interviews were audio recorded, with participants’ permission, and transcribed verbatim yielding 7,225 lines of text. All transcripts were reviewed against the original audio files by a member of the research team to ensure accuracy. Any identifying information was removed from the transcripts in order to protect participant confidentiality.

**Table 1:** **Participant characteristics by gender, geography and type**

		**Clients & informal caregivers (n = 28)**	**Health care providers (n = 20)**
**Urban (n = 16)**	Male	4	0
	Female	8	4
**Rural (n = 32)**	Male	1	1
	Female	15	15

The focus group interviews followed the approach outlined by Krueger and Casey [24], and were facilitated by semi-structured interview guides (Additional file 1). Two separate interview guides were created: one for use with health care providers and the other for clients and family caregivers. In order to reduce issues related to social desirability bias [25], these two participant groups were interviewed separately to maintain group homogeneity. Health care provider groups were further divided into separate sessions: one with community case managers and directors who worked with Community Care Access Centres (CCACs coordinate community care and long-term care placement in Ontario) and the other with additional health care professionals. The diversity of participants allowed the researchers to compare perspectives from different types of individuals on CBPHC experiences [26]. All focus group and individual interview sessions were led by a facilitator and accompanied by at least one note-taker. Saturation occurred after the fifth focus group interview. Two additional focus group interviews took place after saturation and still, no new information came forward.

This study received ethics clearance from the University of Waterloo’s Human Research Ethics Board as well as clearance to recruit and conduct focus group sessions within participating agencies. Informed consent was obtained from all participants prior to the start of each focus group. Each participant was provided with both a written and verbal description of the study as well as the opportunity to ask questions before providing their signature to indicate consent.

### Data analysis

Data were analyzed through a combination of directed and emergent coding [27] using NVivo 10 software [28]. Directed coding was used to categorize the data into three broad domains: barriers to effective CBPHC, facilitators of CBPHC, and recommendations for system improvements [27]. Emergent coding was then used to uncover themes within each domain [27], working from highly specific to more abstract themes. Coding was done individually by three researchers to ensure reliability of findings. Cross-checking was used to highlight any discrepancies or alternative interpretations which were then discussed until consensus was reached [22]. A list of themes and subthemes were then generated for each of the three domains.

As a member check, results were shared with participants through in-depth sessions that lasted approximately one to one-and-a-half hours to ensure that the findings were credible to those who participated in the interviews [29]. Two member check sessions were held with clients and informal caregivers, and one with health care providers. Initial analysis of the key points was provided to participants and their feedback was used to ensure that the list of themes was exhaustive and representative of their experiences.

## Results

Within each of the three broad domains (barriers, facilitators, and recommended system improvements), several themes and subthemes emerged. Although negative care experiences were easily recalled by all participants, they also provided insight into current initiatives that are working well, and innovative ideas for future health care system improvements. Quotes were chosen for inclusion in this article based on their representativeness of the theme they were categorized under as well as their overall impact. A summary of the themes and subthemes is provided in Table 2.

**Table 2:** **An illustration of the domains, themes, and subthemes**

**Barriers**	Poor System Integration	Poor Communication
		Difficulty Navigating the System
		Barriers to Information Exchange
		Lack of Consistency Throughout System
		Inconsistent Follow-Up Care
	Limited Access to Services	Policy and Funding Restraints
		Complicated Specialist Access and Referrals
		Inadequate Transportation
**Facilitators**	Person and Family-Focused Care	Holistic Care
		Inclusion of Informal Caregivers
	Self-Management Resources	Supportive Programs
		Education and Training
		Support Groups
	Successful Collaborative Practice	Team-Based Care Delivery
		Case Conferences
		Providers Who Enhance Patient Experience
**Recommended System Improvements**	Expanding and Integrating Care Teams	Incorporating Diverse Providers
		Inter-professional Collaboration
	System Navigation	Patient Advocacy
		Awareness of Community Resources
	Development of Standardized Assessments, Information Systems, and Care Pathways	Standardized Assessments
		Standardized Information Systems
		Standardized Service Delivery/Care Pathways

### Barriers

#### Theme 1: poor system integration

Lack of communication in CBPHC was a source of frustration for all participant groups and was one of the most frequently mentioned barriers to care. They discussed the lack of dialogue between different providers interacting with the same patient, as well as the common miscommunications between patients and their providers. One urban patient commented on the lack of communication between health care providers:

*‘“It’s not unusual for this day and age to have two or more medical practitioners that you deal with and in my experience they are often medical alpha dogs and getting one to talk to the other isn’t always easy.’”*

An example that was frequently mentioned involved patients who sought care from a hospital Emergency Department. Primary care providers often did not receive any test results or patient information from that visit and in some cases, the provider was not aware that the person had visited the Emergency Department.

Participants discussed how poor communication between health care providers often led to unnecessary repetition of assessments - patients were repeatedly asked the same questions by different providers which caused frustration for both them and their caregivers. Communication issues also existed in the patient-provider relationship. Patients expressed feelings of not being heard or listened to, and informal caregivers often felt left out of important care conversations. One rural informal caregiver discussed how no one had explained the medical procedures that she would be required to perform on her husband once he left the hospital:

*‘“They have a person at the hospital that’s with Community Care. She came in to see my husband three times I believe. But they were dealing with a biliary tube that had to be flushed. And he wasn’t going to be able to do that. So – but they never talked to me and I was the one that was going to do it - so to make the story short, I think that they should zero in on who is going to be doing the procedure when the person goes home and at least go over with them the supplies and the procedure and everything, make sure that that’s going to work.’”*

Difficulty navigating the health care system was another barrier discussed in all of the focus group interviews. The current health care system was described as complex, confusing, and difficult to navigate for patients and informal caregivers. Patient and caregiver participants described feeling overwhelmed by the number of providers who were involved in their care. Both patient and provider participants described the difficulty that patients had with keeping track of ‘who was doing what’ and knowing which service to contact when they experienced difficulties. As one rural patient explained:

*‘“It was just keeping track of it all. It would have been nice to have just one [phone number] but you had to have all different phone numbers, all different people.’”*

Providers, especially those in urban areas, were not always well informed about the community resources that were available for their patients. They found it difficult to keep track of which services were available and to whom, since there is currently no central database to consult for current programs and initiatives targeted to seniors. In rural areas where the number of services was limited, providers were more aware of the services to which they could refer their patients. Navigation issues were deemed particularly relevant during patient transitions from one care setting to another (e.g., discharge from hospital to home). Often patients and their caregivers felt rushed and unprepared for the transition. One rural patient describes his experience being discharged from a hospital-based rehabilitation program:

*‘“During the three month stay every time someone - it was interesting that, like you know, everybody would say, ‘Well when are you going home?’ Well no don’t know yet, they don’t know yet, you know nobody knows you know and then all of a sudden one day they come in and say tomorrow you’re going home or even, yeah usually tomorrow or even the same day you’re going home and nobody from the CCAC*^*a*^*has been in to set anything up until the morning of the, like two hours before discharge and sometimes they weren’t even, and people were supposed to get their own equipment and supposed to, you know, do all these things, and if they didn’t have family or their family or, you know, wasn’t available right then, I mean it was a really stressful situation for them because they were panicking because they didn’t know what they were supposed to be doing or who was going to look after them or what agencies they were going to need.’”*

There were also obstacles relating to poor information flow between those involved in a patient’s care experience. One factor preventing full information exchange between patients and providers was the limited appointment time that certain providers had with patients. One rural patient described the difference she saw with shorter appointment times amongst various providers:

*‘“And there’s not enough time to spend with the patients. There’s sometimes you really think, ‘oh, mine’s not that important’, so you quickly go state your things, get me my pills or whatever, you know, and that whereas you’re not – but with the nurse practitioner you’re more relaxed and you will chat more with them, but with the doctors you seem, you know, and you go to a specialist. Well, he’s a busy guy. Look at how busy they are.’”*

Other themes that emerged from the data indicated a general lack of consistency within CBPHC settings. Large differences between the care provided by solo practice physicians versus a team-based primary care setting, such as a Family Health Team (FHT) or Community Health Centre (CHC), were noted. For example, provider participants noted the difficulty of engaging solo practice physicians in new initiatives, claiming that physicians working as part of a FHT were much easier to keep informed. One urban provider commented:

*‘“FHT is still much easier. It’s the individual family physicians who are not connected. That becomes an issue.’”*

Services offered in different geographical regions were also inconsistent, making it difficult for patients to access nearby resources. Often patients who lived close to the boundary of two different regions were geographically closer to the services of the adjacent region, and not the region in which they officially resided. However, because of these strict geographical divisions they were only able to access the resources from the region in which they resided. Both patients and providers mentioned this issue. One urban case manager commented:

*‘“So, sometimes for rural areas, for example, it becomes very difficult to access any of the services and there is a very funny situation which happens when the location actually is on the border of other regions or other CCAC – that’s true for CSS*^*b*^*also. It is much more faster if they get services from the other CCAC instead of us but since that’s in the so-called area jurisdiction, whatever you call that, they cannot do that.’”*

Finally, participants discussed the lack of follow-up care initiated by health care practitioners. A common example involved patients who had visited the Emergency Department; since their providers were often not notified of the visit, follow-up was not initiated. Patients felt that they should not have to initiate their own follow-up appointments; however, providers stressed that they did not always receive complete patient information, thus it was necessary for patients to actively seek follow-up care. This difference in patient and provider expectations frequently led to a disjointed care experience for the patient, and frustration for both sides.

#### Theme 2: limited access to services

Policy issues and funding restraints were repeatedly mentioned as reasons that providers were not able to provide the holistic care that both they and their patients desired. For example, current funding reimbursement models in Ontario discourage family physicians from practicing preventive care and home visits, since the compensation provided for these services is minimal compared to medical interventions provided in a clinical setting. One urban patient commented:

*‘“But that requires a change in the structure of the OHIP*^*c*^*remuneration system. Because a doctor who visits someone at home, it will take that doctor five times as long than if the doctor was in her office – she could see five patients in that time and she would get paid five times as much. So the OHIP and the other provinces have got to restructure their system if any system of home visiting is going to have any hope of success.’”*

Other obstacles included the limits placed on funding for services deemed ‘“non-essential’” by provincial health insurance, such as physiotherapy. One urban patient explained how she exhausted her physiotherapy coverage before her treatment was complete:

*‘“If you come home from surgery – I’ve had two knees done, the physiotherapist has come to the house. Privately. But it only goes for so long and then it’s not covered by OHIP, so if you still need more beyond that, you have to pay for it or do without. It’s kind of a disconnect because there’s no one there to say ‘oh, you need more and OHIP will pay for it’. Or ‘you don’t need more’. So we’re on our own at that point.’”*

Lengthy wait times and confusing referral systems were both described as significant barriers to care, especially for those attempting to access specialist services. Participants described experiencing long wait times for specialist appointments in addition to having trouble scheduling appointments at a convenient time. For example, rural participants discussed the difficulty of having a mid-day specialist appointment; these appointments were often only available in urban locations, so it required taking an entire day off work to be able to travel to and from the specialist in the middle of the day. Many also mentioned the time restrictions placed on referrals and the need to go back to primary care for a second referral in order to have more than one consultation with the same specialist. One rural patient with previous work experience in the health care field said:

*‘“I worked in multi-doctor’s offices in my time, like most of my career, and if somebody needed to see a specialist, one phone call did it. You had the appointment and the person was on their way. I found out yesterday the process here is they write a letter. Has to be signed by the doctor. Then they fax it to the specialist. The specialist has to fax the appointment back to the health care team here. Then they phone me. Now that, to me, is absolutely ridiculous.’”*

Transportation was repeatedly brought up as a barrier for access to CBPHC, particularly in rural areas where there is no available public transit. Even if high-quality health care services were offered in the area, seniors and their caregivers who lived outside of the town centre were not always able to use them. One rural informal caregiver who volunteered as a driver for older adults said:

*‘“Personally what I’m most involved with there is the transportation when needed because we live in a rural area. It’s a major factor for a lot of the seniors and patients, if you will, because they are so sick they can’t drive and so if family members are not available to drive them then there’s limited choices.’”*

### Facilitators

#### Theme 1: person and family-focused care

Being viewed as a person and not merely as an illness was a common theme throughout the focus group discussions. Patients and informal caregivers expressed appreciation for receiving care that fit their needs and for working with providers who took the time to see them as a person. They also praised providers who tailored treatments specifically to their individual situations. Both patients and providers stressed the importance of holistic care and being able to take the unique patient context into account when providing treatment. One rural provider described the person-centered approach that their CHC used to work with patients:

*‘“So I guess for myself I think it’s very important to find out what the patient’s goals of the appointment are, so instead pushing on my goals to the patient I think it’s important to know what their expectations are in the appointment and address those issues before pushing on my ideas.’”*

Both patient and caregiver participants discussed how they valued the involvement of caregivers and family members as active participants in the care process and discussions surrounding care. This was noted to be of especially high importance during care transitions when a lack of coordination or information flow affected the patient’s care experience. One rural caregiver described a positive care experience she had with a primary care provider:

*‘“I really appreciate being included as a caregiver as part of that discussion and we’re really fortunate, you know, all the specialists, you know, will go through their whole thing and then they’ll turn to me and say, ‘oh, so do you have any further questions?’ after they’ve checked with my husband. But I really find that so valuable because it increases my comfort level with being a caregiver and making sure that I’m understanding fully what is going on.’”*

#### Theme 2: self-management resources

Participants remarked on the value of current education and training initiatives for patients and caregivers. They discussed how self-management support groups and resources allowed patients to be more engaged in maintaining their own health and helped to prepare them for discharge or care transitions. Getting to know other patients with similar health conditions also proved to be a good source of social and emotional support.

All participants were able to list resources within their communities that they felt were providing valuable supports for self-management. These included day programs for seniors, exercise classes, and healthy eating programs. One rural provider elaborated:

*‘“Yeah, no, I think we’ve got some really good programs,[theCentre] offers a lot of different senior exercise programs, dining program, the adult day centre. They’ll now come out into the home and set up an exercise program for people, which is great just to try to keep them as active as they can be.’”*

Another example was a coffee hour hosted by a local hospital once a week for people with Alzheimer’s disease. It provided an opportunity to socialize with people experiencing similar situations while providing a break for their caregivers.

One program mentioned in several focus groups was the CCAC-run Integrated Assisted Living Program (IALP). This program targeted areas with a high concentration of seniors, which also tended to be areas of lower socioeconomic status, and offered localized specific services to meet their needs (e.g., meal delivery). For example, if there was a single apartment building that had several IALP clients located within it, services and programs would be offered directly to those clients within the building.

#### Theme 3: successful collaborative practice

Team-based models of primary care delivery, such as FHTs and CHCs, were mentioned as successful examples of providers working collaboratively. These practice models make it easy for patients to be seen by multiple providers and also allow providers to collaborate on shared patients. Participants described these models as providing a holistic care experience to their patients.

Several health care providers discussed holding case conferences for complex patients as a way to coordinate with everyone involved in the treatment process. Case conferences gather together the patient, their caregivers, and all of their formal health care providers in one room to discuss the current treatment plan and next steps. One rural provider explained:

*‘“I think the CCAC seems to be having more case conferences. And I think that’s an excellent opportunity to coordinate our care so that we know that we’re all on the same page and we’ve got the client’s goals and you know, that everybody’s working together with this.’”*

Participants commented on several health care provider roles that currently work well within the system to enhance patient experiences. The most commonly cited example was the role of nurse practitioners. Both providers and patients enjoyed working with them and believed them to be valuable members of the health care team. Geriatric specialists and case managers were also cited as positive examples.

### Recommended system improvements

#### Theme 1: expanding and integrating care teams

Participants proposed expanding primary care settings to include additional diverse providers that could help alleviate the workload placed on physicians and offer a more comprehensive set of services. Suggestions included implementing nurse practitioners, system navigators, physician assistants, qualified international doctors, and care coordinators to improve patient experiences while maximizing health care resources. One urban patient discussed nurse practitioners as the first touch point of care for people with minor ailments:

*‘“So if somehow the ordinary doc who’s working on his own or with one or two other people, could get some funding for a nurse practitioner, and if the nurse practitioners were available, that would be a great first line of defence and hopefully you wouldn’t have to wait for a sore throat.’”*

However, it was also noted that to achieve inter-professional collaboration, all of these primary care providers would need to be further educated on each other’s roles to understand how their scope of practice would fit within the larger care team in order to operate efficiently. One rural provider mentioned a previous educational initiative as a potential solution:

*‘“Each one of the different therapies and the nurses and the different people took the… took a typical day and sort of said what they did in a typical day so that you know they had a much greater respect for what the OT* (occupational therapist) *did and so if you saw an issue out in the community, you knew that you know, and OT* (occupational therapy) *would help them so much here. They really helped.’”*

#### Theme 2: system navigation

Support was expressed for the role of patient advocates, who could help the patient navigate the care system and enable them to participate more fully in decision-making as part of the team. Participants felt that having someone accompany a patient while they are visiting their health care providers to ask questions, to vocalize the needs of the patient and to be there when information is presented, would be helpful. It was explained that the amount of information you receive as a patient is often overwhelming, and having someone there to help keep track of everything would ensure optimal information exchange. The example was given of a patient who receives a cancer diagnosis - often they could not absorb subsequent information after hearing the diagnosis. A patient advocate could gather additional information from the health care provider and go over it again with the patient at a later time. This would be especially important for patients who do not have a family member that they could bring along to appointments. One rural patient described what the position might look like:

*‘“I would like to see a position of a patient advocate in the hospitals. And that would then - they would know when to bring the CCAC and they would know, you know, when they should be looking at different types of care or know what the situation is at the home. And especially if there’s no family or anybody – if they’re – if they don’t have anybody to speak for them, I think that would be a really – that’s what I would like to see.’”*

Although several examples of supportive health promotion programs and initiatives were cited (as detailed under ‘Facilitators’), participants described a general lack of awareness among community members of available resources. It was discussed that better advertising and promotion of the resources offered in different communities would increase participation in health promotion and self-management activities.

It was also recommended that primary care providers stay up-to-date on the community resources available to their patients so that they could knowledgeably refer patients to relevant services or activities. In the same vein, it was suggested that agencies also make efforts to make providers aware of the services they offer. One rural patient discussed the importance of providers being fully informed:

*‘“More knowledge about what’s available and all, and the knowledge, if you will, of what’s available more generally spread across the different caregiver types of things so that, someone comes in to see the doctor, their people there are more aware of saying, ‘well if you need transportation like there’s this number and this available here, this is available there, that’s available there’.’”*

#### Theme 3: development of standardized assessments, information systems and care pathways

Many participants noted the importance of both standardized practices and standardized information tools. A routinely employed set of assessment tools was recommended as a solution to the redundant questions asked by different providers. This may help to provide basic information to everyone involved in the care process, allowing them to save time during an appointment and focus on questions relevant to their area of expertise.

Participants recommended the implementation of a common health information system so that providers could access up-to-date patient information. This was suggested as a solution to illegible handwritten notes in patient charts and problems with incompatible software being used by different providers.

In order to maintain a consistently high level of care across settings, one recommended system improvement was to standardize service delivery within a profession. One urban provider elaborated:

*‘“And in terms of the same way that nursing delivered from one agency to another agency needs to be the same. That you can expect this initial assessment and this to happen and this to happen, but until there’s some standardization, I think it’s hard to say that we’re going to be client-focused or we’re going to get the physicians on board, when, you know, within the network at this time there is not good enough standardization. And that sort of flies in the face of being client-centred and everything is individual for the client but it’s the – I think probably the information or the method in which we deliver service -sometimes that really needs to be standardized and that way people get to know what to expect.’”*

Additionally, the development of care pathways was recommended as a way to standardize the treatment process and to keep patients and providers on the same page. For example, one urban provider participant suggested the creation of a chronic care management template:

*‘“Like why don’t they – physicians - have that template to be able to use, you know, then I thought ‘no, really!’ And it has been sort of my imagination but I keep imagining that they would have that on their computer screen and when that person came in, they just plugged in that person’s chronic disease and they would know, well, yes, they’re due for their blood sugar testing and yes, their foot care is due now and, you know, I keep imagining that they would use a chronic disease management template to make it happen.’”*

## Discussion

This study identified current barriers, facilitators, and recommended system improvements for CBPHC for older adults, from the perspectives of patients, informal caregivers, and health care providers.

Identified barriers to improved CBPHC included: poor communication between patients and providers, challenges in navigating a complicated health care system, roadblocks to information exchange, a general lack of consistency in service delivery, inconsistent follow-up care, and policy and funding constraints. The barriers identified by urban and rural participants were similar with the exception of transportation being discussed solely by rural participants. Complicated specialist access and referrals was a theme common to all participants, but rural patients and caregivers focused on the difficulty of mid-day out-of-town appointments, while urban participants spoke more about lengthy referral wait times. Overall, these findings are consistent with current literature that has identified both poor system integration [14,17,19,30-32] and access, particularly access to specialists, as barriers to health care in Canada [30,31,33-36]. We note, however, that the Health Council of Canada [32] found that access to primary care was actually higher for persons with chronic illness when compared to members of the general population, perhaps indicating that health care providers are prioritizing these patients with higher needs. While this may be true, our study suggests that additional efforts to facilitate access to CBPHC for adults with chronic illness are still needed.

Three themes relating to facilitators in primary care were identified in this study: person and family-focused care, self-management resources, and successful collaborative practice. There were no significant differences between the facilitators identified by urban and rural participants. Person and family-focused care, commonly referred to as patient-centered care, has been identified as a key priority for the Canadian health care system [30,31,37,38] and has been shown to increase care efficiency and improve patient health outcomes [39]. This study found support for increased resources for self-management, consistent with reports that engaging patients in their care leads to increased positive feelings about their health [35] and that peer support can increase patient self-efficacy [40]. While self-management has been described as an essential aspect of care, particularly for individuals with chronic conditions [40], other research has found a limited impact of self-management interventions on health outcomes and system utilization [41,42]; methodological limitations of some studies suggest additional research is warranted [42].

Successful collaborative practice as a current health care facilitator is congruent with current literature and research findings [31,33,36,38]. Team-based care, as an example of successful collaborative practice, is increasingly common and some evidence exists indicating that it can improve clinical outcomes [43]. Poulton and West [44] found that having clear objectives that team members were highly committed to accounted for a large portion of the varying levels of effectiveness amongst primary care teams. This suggests that truly collaborative care extends beyond housing diverse health care professionals under the same roof, since more providers in the same environment does result in increased organizational complexity [45]. Education on how to collaborate and work alongside other health care professionals is an essential component of team success [46].

Participants identified three categories for system improvements to address barriers and enhance the positive aspects of the current health care system, with no significant differences emerging between the urban and rural participants. The first theme, ‘expanding and integrating care teams’, aligns with the previous discussion of collaborative practice as a CBPHC facilitator and current strategic directions addressing the implementation of community-based care teams composed of diverse providers [31,35,47].

The second theme related to resources that would help complex older patients to navigate the health system. A recent literature review by Manderson and colleagues [18] found evidence in support of a system navigation role for older adults with chronic illness, but also identified a need for further development and clarification of the most effective navigation roles and approaches. Participants further recognized that despite a wide array of health promotion community initiatives, both older adults and their providers were not consistently aware of the available resources. Bodenheimer and colleagues [48] described linkages between clinical settings and community health resources as highly important, particularly for health care professionals who are not operating as part of a large team-based organization and for those treating patients with chronic illness. Clinicians that are aware of the appropriate programs to refer their clients to could be an important tool for increasing participation in these initiatives.

The third theme focused on the standardization of clinical practices, health assessment and information systems, and pathways. Creating consistency through standardization is often addressed in the literature through health information technology which will improve care integration and promote patient safety [31,47]. Electronic health records are a critical component of integrated primary care and care management, since they facilitate the flow of important patient information amongst the providers involved in the circle of care [38,49]. Previous research has shown the lack of interoperability between different electronic health information systems to be a significant barrier to informational continuity [50]. McMurray and colleagues [49] performed a large multi-site ethnographic study investigating care transitions for older adults and found that most of the health care professionals involved were using a combination of electronic and paper charting which increased, rather than lessened, the demands on their time. The slow transition to electronic health records in Canada was shown to result in high levels of information duplication for a range of providers, including both solo-practice and hospital-based physicians [49]. A systematic review by Fontaine and colleagues [51] showed evidence for electronic health information systems as a way to improve patient safety and reduce medical errors, as well as a way to ‘“improve access to test results and other data from outside the practice, to improve referral processes and claims processing, and to decrease staff time required for handling these processes.’” Thus, while the benefits of having a standardized electronic health system have been illustrated; further work is needed to facilitate its full implementation.

These findings reflect the views of patients over the age of 65, their informal caregivers, and their formal care providers, highlighting both areas of the system that are currently working well, and areas that will require further improvement. Older adults as a population are heterogeneous and complex to care for because of high rates of chronic illness and multi-morbidities [52]. An integrated and accessible system of care, while important for all older adults, would be especially critical for those with chronic diseases who are at increased risk of polypharmacy, mortality and other adverse outcomes [52]. The themes that emerged from these data correspond with several domains from Wagner’s Chronic Care Model which highlights the importance of community resources, health care organization, self-management supports, delivery system design, decision support, and clinical information systems in the management of chronic disease [53,48]. Our findings reinforce the value of chronic care models as frameworks to guide health system improvements, as well as the need for multi-faceted and integrated approaches. Although our work did not specifically identify themes related to the community action component of the Chronic Care Model, strong community partnerships would be essential for any system reform effort.

### Limitations

This study has several limitations. Focus group interviews were held in community settings which likely prevented very ill patients from attending and thus the results may more heavily reflect the perspective of healthier older adults. Including caregivers for adults over the age of 65 may have mitigated this effect as the caregivers would have been able to reflect on the experiences of more seriously ill patients for whom they care. Also, one urban patient/caregiver focus group was held directly within a retirement community to make it easier for less mobile patients to attend. Of the 48 participants interviewed, a larger number of participants (n = 32) were from a rural setting compared to an urban setting (n = 16). As a result, study findings may over-represent the views of this group. This study was conducted within the Canadian province of Ontario; while we believe results will be relevant to other publicly-funded health systems, unique features of other systems would need to be considered in applying our findings in other settings. A mix of urban and rural locations was included to maximize generalizable and include a variety of perspectives.

## Conclusions

Older adults are not well served by the current health care system and with aging populations in Canada and many other jurisdictions, creating a more accessible and integrated system is an urgent priority. At the system level, policies are needed to support the integration of primary health care services so that a truly system-wide shift can occur. This study allowed for consensus on the barriers, facilitators, and areas for improved system integration with respect to older persons accessing CBPHC. These results can be used to inform primary health care reform at multiple levels, so that future initiatives reflect the views of the stakeholders who are directly involved in the care process. Future interventions should include the expansion and integration of interdisciplinary primary health care teams, patient advocacy and system navigation, the implementation of standardized assessments, information systems and care pathways, and the development of effective self-management supports for older adults. Additional work is needed to implement the recommended system improvements identified by participants and to discern their outcomes and effects on CBPHC as a whole.

## Endnotes

^a^Community Care Access Centres coordinate community care and long-term care placement in Ontario.

^b^Community Support Services (such as ‘“Meals on Wheels’”).

^c^Ontario Health Insurance Plan, which provides coverage for publicly-funded health services in Ontario.

## Additional file

Additional file 1:
**Contains two interview guides that were used to conduct the focus group interviews: one guide for clients and families, and a separate guide for case managers, service providers, and administrators.**

## Abbreviations

CBPHC: Community-based primary health care
CCAC: Community Care Access Centre
CHC: Community Health Centre
CSS: Community Support Services
FHT: Family health team
MOHLTC: Ontario ministry of health and long-term care
OHIP: Ontario Health Insurance Plan
IALP: Integrated assisted living program
OT: Occupational therapy/therapist
