# Supplementary material for: Community-based primary health care for older adults: a qualitative study of the perceptions of clients, caregivers and health care providers
Source: BMC Geriatr. 2015 Apr 30;15:57. doi: 10.1186/s12877-015-0052-x (PMC4434544; doi:10.1186/s12877-015-0052-x)
Supplement: Additional file 1: — Contains two interview guides that were used to conduct the focus group interviews: one guide for clients and families, and a separate guide for case managers, service providers, and administrators. [file 12877_2015_52_MOESM1_ESM.pdf]

Additional File 1: CBPHC Planning Grant Focus Group Interview Guide Questions (Clients and Families)

<u>Questions:</u>

1. Who has been a recipient of primary health care in the last month?
2. Who has been a caregiver to someone who has received primary health care in the last month?
3. What are some of the words you would use to describe "quality of care"?
4. Can you give an example of a time when you feel you received "quality of care"?
5. What is one reason for this being a positive experience for you?
6. Can you give an example of a time when you feel you didn't receive "quality of care"?
7. What could have improved this experience to make it more enjoyable?
8. What does "patient centred" care mean to you?
9. What are some improvements that could be made to service delivery to make it more "patient friendly"?
10. What do you think are examples of ways to promote a more active and healthy aging community? How can we engage/empower seniors to become more involved in their own care?
11. How can the system better support individuals who are trying to manage their own care?
12. How could the system be improved to make the care you receive more seamless?
13. If you were asked to come up with a value statement to put up in your doctor's office about the care that you expect to receive, what would it say?
14. If you had 1-minute to talk to an influential decision-maker about the care you have received (or improvements that need to be made to the health care system) what would you say?

Additional File 2: CBPHC Planning Grant Focus Group Interview Guide Questions (Case Managers, Service providers, and Administrators)

<u>Questions:</u>

1. What does patient-centred care mean for different people and families?
2. What are some improvements that could be made to service delivery to make it more “patient friendly”?
3. What do you think are examples of ways that we can promote a more active and healthy aging community?
4. How can we empower seniors in their care?
5. How do you build capacity in communities for integrated care?
6. What is the role of geriatric specialists?
    a. What are the benefits and strategies to involving them early?
7. Improving linkages across the system has been identified as a focus for our region for integrated care. What linkages are most important and how should they be targeted? (consensus)
8. What roles support care transitions, what is missing?
9. How can we provide long term, low intensity support to individuals who are at risk of deterioration?
10. What changes need to be made to 1) roles, and 2) tools/processes to support system navigation?
11. How can primary care adopt even more of a proactive, preventative approach?
12. What successes have you had in communicating and collaborating with other professionals?
13. How can we better support individuals who are trying to manage chronic conditions?
14. If primary care were the anchor for care coordination, what might that look like? What are some examples of successes from other sectors that can be used as a model?
